# Physiotherapeutic Rehabilitation of a Patient Following an Electrical Burn: A Case Report

**DOI:** 10.7759/cureus.29702

**Published:** 2022-09-28

**Authors:** Akanksha R Hege, Chitrakshi A Choubisa, Pooja Kasatwar

**Affiliations:** 1 Physiotherapy, Ravi Nair Physiotherapy College, Datta Meghe Institute of Medical Sciences, Wardha, IND; 2 Community Health Physiotherapy, Ravi Nair Physiotherapy College, Datta Meghe Institute of Medical Sciences, Wardha, IND

**Keywords:** contracture prevention, burn physiotherapy management, burn contracture, burn management, electrical burn

## Abstract

Electrical burn is one of the common burn injuries occurring nowadays due to the increase in the use of technology; among others, electricians are more prone to electrical burn injury as they work all day to make and repair electrical equipment and systems. In this case report, we are presenting such a scenario. The patient, a 36-year-old male, was brought to casualty with an injury by a flash of electricity while working. The patient sustained a burn on the right hand and presented with burn injury over the right hand and forearm with bleb over the anterior aspect of the wrist joint, skin discolouration, local rise in temperature, line of demarcation seen over the palmar aspect of the forearm at middle 2/3^rd^, clear serous discharge present from bleb, discolouration of tips of all fingers of the right hand and nail beds, and tenderness. The patient was diagnosed with an electrical burn injury after the evaluation and investigations. The investigations were done on complete blood count (CBC), coagulation profile, peripheral smear, liver function test (LFT), and random blood glucose (RBS). The injury site was cleaned and the dressing was done with one percent silver sulfadiazine, some medications were prescribed by the physician, and plaster was applied to prevent contractures. The physiotherapeutic intervention for the prevention of burn contracture includes positioning, splinting, massage, stretching, scar management, pressure therapy, and strengthening. This case report concludes that early physiotherapeutic interventions helped in the prevention of electrical burn contractures and the patient’s functional mobility.

## Introduction

Burn-related injuries are the most devastating experience that an individual can face, which have various secondary complications associated, including infection, pulmonary complications, metabolic complications, cardiovascular complications, heterotopic ossification, neuropathy, pathological scars, and contractures [1,2]. Burn injury may be caused by fire, hot liquid, steam, hot object, ionizing radiations, electric current, chemicals, cold injury, and sunburns [3]. In this case report, we have discussed the burn injury which is caused due to electric current. When an electric current comes in contact with the body due to which skin burns causing electrical burn injury, the body converts the electric current into heat which causes thermal burn injury. A burn injury depends on the percentage of burn and the skin thickness involved. According to the percentage of burn, it is classified into mild/minor, moderate, and major/severe, whereas according to the thickness of skin involved, it is classified as first, second, third, and fourth degree of burn [4]. Patients with burn injuries must undergo extensive, interdisciplinary therapy beginning the day of their accident. To lessen the impact of the patient's traumatic experiences and increase functional independence, a thorough rehabilitation program is needed [5]. Various physiotherapy interventions have shown improvement in primary and secondary complications. With a pain perception reduction of more than thirty percent, virtual reality distraction seems to be most beneficial for patients with hypersensitivity to pain [5,6]. Second and third-degree burns lead to the tightening of the skin and formation of scars, which may lead to contractures and manifest as pathological scars. To prevent this, anti-contracture positioning, splinting, and pressure dressing should be included in early physiotherapeutic rehabilitation [7,5]. Stretching and strengthening play an integral part in maintaining muscle physiology and joint mobility [8].

## Case presentation

Patient information

A 36-year-old male, working as an electrician, came to the casualty with a history of injury by a flash of electricity while working at his workstation, at 12:15 pm on January 16, 2021. The patient had sustained a burn on the right hand; there was no history of injury elsewhere on the body. The patient was presented with redness, tenderness, bleb over the anterior aspect of wrist joint with clear discharge from it, discolouration of the skin, local rise in temperature, and a line of demarcation seen over the palmar aspect of the forearm at middle 2/3rd. He was advised for rehabilitation of the hand. The patient’s medical, family, and psycho-social history was not significant.

Clinical findings

**Figure 1:**
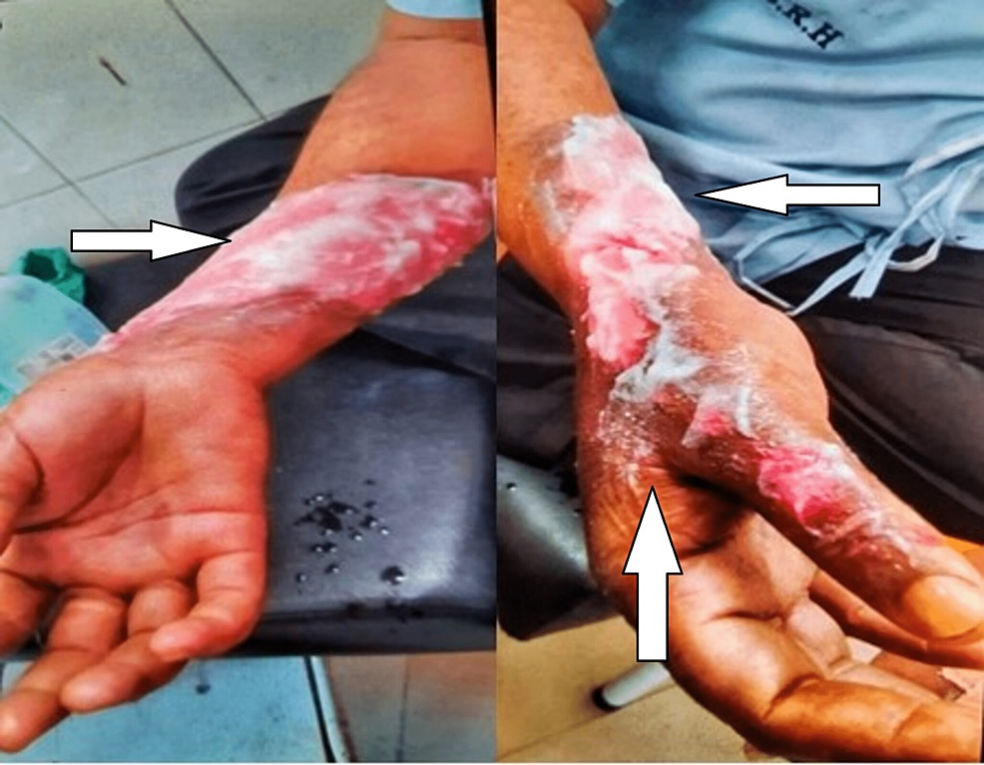
Presentation of the patient’s extremity on the day of the burn

Consent was taken before the examination. He was observed while sitting with both shoulders at the same level. On examination, burn injury over the right hand and forearm was found present. There was discolouration of the skin, tenderness grade three, and redness present. Oedema was also present over the forearm, wrist, and fingers. The percentage of total body surface area burned is calculated by Wallace’s rule of nine. The patient had a 3.5% of burn and it was a deep partial thickness burn that was a second-degree burn in which damage extends through the epidermis and involves the dermis (Figure 1). On Numerical Pain Rating Scale, the patient rated pain intensity as 8/10. On sensory and motor assessment, no abnormality was found. Respiratory, and cardiovascular systems were normal.

Physiotherapeutic management

**Figure 2:**
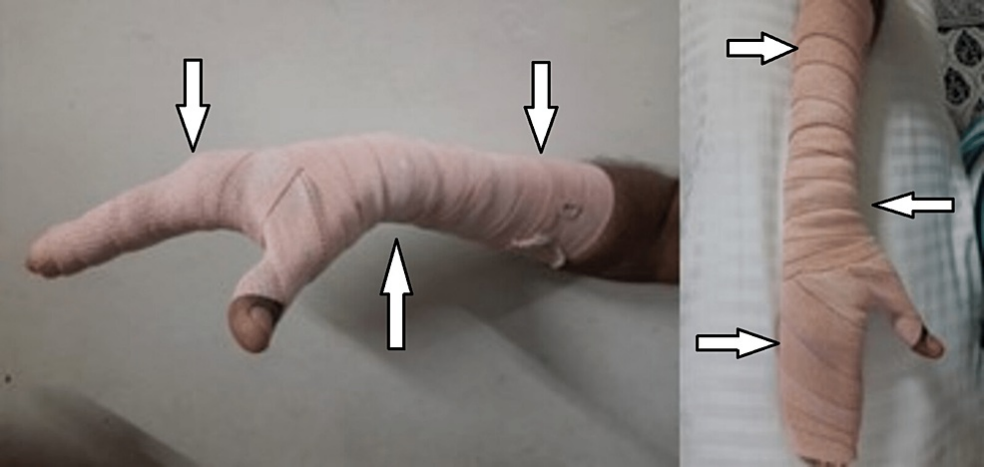
Positioning with pressure therapy using crepe bandage

**Figure 3:**
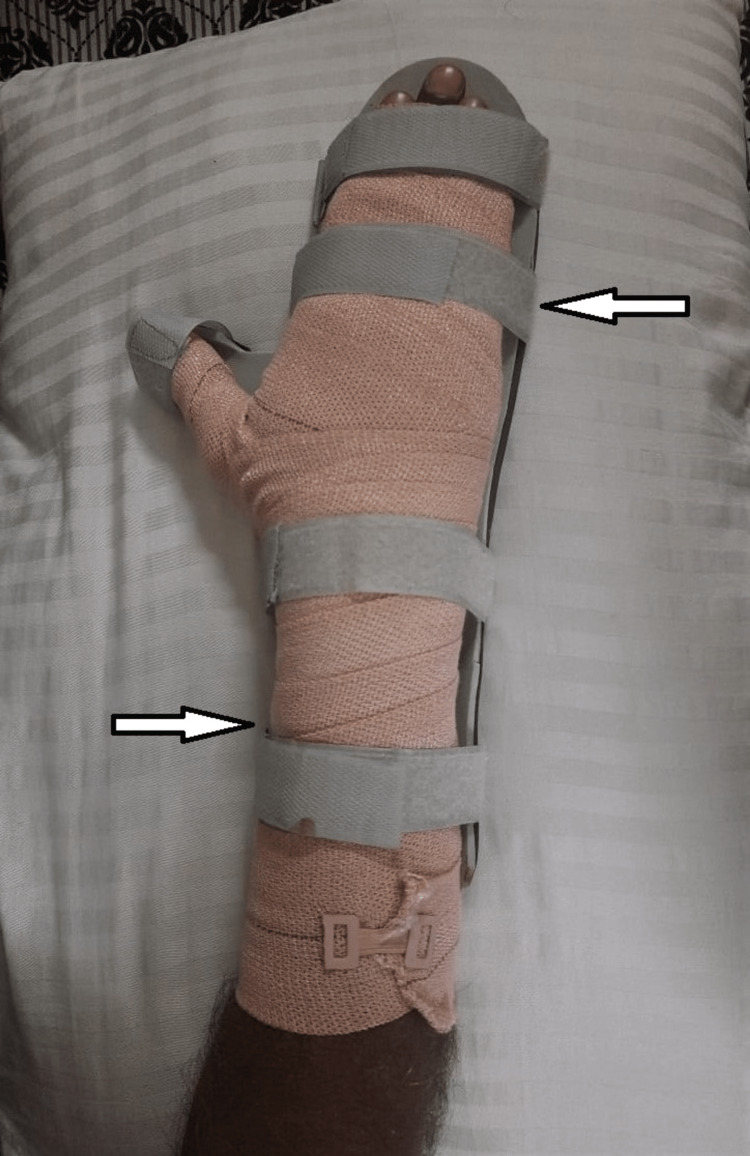
Splinting with cockup splint

Early physiotherapeutic rehabilitation was initiated, which mainly focus on pain relief, positioning, and splinting to prevent contracture, stretching, and early mobility to make the patient independent and normalize the activity of daily living. A well-structured home care program was discussed with the patient along with its implementation in his daily routine. The rehabilitation program has been discussed in Table 1. Positioning with pressure therapy using a crepe bandage is shown in Figure 2. Splinting with cockup splint is shown in Figure 3.

**Table 1:** Rehabilitation program AROM=active range of motion; PIP=proximal interphalangeal; DIP=distal interphalangeal; MCP=metacarpophalangeal; ROM=range of motion

Goals	Intervention	Rehabilitation program
To reduce pain and improve patient participation	Virtual reality	The use of virtual reality during the physiotherapy intervention to distract the patient from pain perception, thus improving the cooperation and participation of the patient
To prevent contractures and maintain muscle elasticity	Anti-deformity positioning, splinting, and stretching	Positioning and splinting using cockup splint were given continuously for initial 2 weeks. Stretching was provided from the third week: three reps x five-second holds. The fourth week: five reps x 10-second holds (Figures 2 and 3)
To control oedema and prevent formation of hypertrophic scar and keloid	Pressure dressing	For one hour, four times a day, from the third week of rehabilitation (Figure 2)
To improve the ROM of the elbow joint, wrist joint, first MCP joint, and interphalangeal joints	AROM exercises	Week 0-2: AROM exercises of the elbow, PIP and DIP joints, 10 reps x two sets, thrice a day. Week 3-4: AROM exercises of the wrist, first MCP joint, and interphalangeal joints, 10 reps x one set, thrice a day. Active-resisted ROM exercises of the elbow, PIP and DIP joints, 15 reps x two sets, thrice a day
To improve the strength of wrist flexors, extensors and intrinsic muscles	Strengthening exercises using dumbells of various weights, silicon ball	Strengthening initiated from the third week of rehabilitation. Week three: wrist flexors and extensors strengthening using dumbbell: 10 reps x one set, Intrinsic muscles strengthening using silicon ball: 10 reps x one set
To prevent adherent scar formation	Scar mobilization	Initiated from the fourth week of rehabilitation for two minutes x five times a day
To improve activities of daily living	Training the left hand for activities of daily living such as grooming, eating, drinking, and gripping	Follow for 2-3 weeks
	Imitating the activities of daily living such as grooming, eating, drinking and gripping	From the fourth week onwards

Follow-up and outcomes

Follow-up was given for three weeks, when the patient was hospitalized and then follow-up was advised thrice a week with regular home exercise training including splinting, pressure therapy, stretching, and strengthening with the prescribed dosage. Outcome measures include pain grading, which was 06/10 after three weeks and after six weeks, it was 02/10 rated on the Numeric Pain Rating Scale. For, range of motion (ROM) refer to Table 2. The patient-Specific Functional Scale (PSFS) rates patient capacity in activities like grooming, holding a pen, eating, and grasping. PSFS score after three weeks was 05/10 and after six weeks, was 09/10. Early initiation of physiotherapeutic rehabilitation resulted in a speedy recovery and early functioning of the limb.

**Table 2:** ROM post physiotherapy management after third and sixth weeks ROM=range of motion; MCP=metacarpophalangeal; IP=interphalangeal

	Follow-up after three weeks			Follow-up after six weeks	
	Active ROM Right	Passive ROM Right		Active ROM Left	Passive ROM Left
Wrist flexion	0-55^0^	0-66^0^		0-79^0^	0-84^0^
Wrist extension	0-36^0^	0-45^0^		0-62^0^	0-66^0^
Ulnar deviation	0-17^0^	0-22^0^		0-24^0^	0-37^0^
Radial deviation	0-11^0^	0-15^0^		0-13^0^	0-23^0^
MCP joints:					
Flexion	0-56^0^	0-67^0^		0-83^0^	0-87^0^
Extension	0-10^0^	0-14^0^		0-24^0^	0-26^0^
IP joints:					
Flexion	0-84^0^	0-97^0^		0-105^0^	0-117^0^
Extension	84^0-^0	97^0^-0		105^0^-0	117^0^-0
MCP joint of thumb:					
Flexion	0-44^0^	0-52^0^	0-56^0^		0-65^0^
Extension	44^0^-0	52^0^-0	56^0^-0		65^0^-0
IP joint of thumb:					
Flexion	0-46^0^	0-60^0^	0-81^0^		0-86^0^
Extension	46^0^-0	60^0^-0	81^0^-0		86^0^-0

## Discussion

Electrical burn injury occurs when there is the passage of electric current through the body which causes burn injury. Every burn injury is followed by the formation of contracture as the wound heals but these contractures may lead to restricted joint mobility, decreased ROM, and the formation of deformity; these complications affect functional mobility. The prevention of contracture is a must for all patients with burn injury as it helps the patient gets back to their normal life. In this case report, we have discussed it.

Electrical burn injury is common in electricians due to contact with power lines at the workplace [9]. The analysis states that the percentage of electrical burn injuries is higher, especially among electricians; it also states that electrical burn injuries are more common in the upper extremity; both are true in the report we have discussed of the electrical burn of the right hand of the patient who is an electrician by occupation [10]. To make patients independent post-burn prevention of contracture is important so that the patients do not have functional restriction, and management of contracture prevention allows early mobilization [11]. The primary way to prevent contracture is to place the affected part in an anti-deformity position which is done with splinting, positioning, and compression therapy [5]. In this case report, we have aimed at the same concept primarily. We have also used the stretching protocol, which was very effective in achieving joint ROM, and this article also stated that stretching remains the key goal for the prevention of burn contracture [12].

Burn scar contracture development is a pathological condition. Prevention of contracture should be focused on in every post-burn patient; adequate positioning and splinting are designed to achieve optimal ROM [7]. The Patient on a splint helps to stretch the impaired fingers and regain ROM; active, active assisted, and passive ROM exercise for the wrist, fingers, and thumb helps in the prevention of contracture and deformity [13]. Strengthening helps in restoring muscle strength using one repetition maximum (RM) and is effective for patients for their functional mobility after achieving full ROM [14]. Scar mobilization is effective for scar prevention as mentioned in a survey done based on clinical experiences of massage therapists [15]. Physiotherapeutic management for the prevention of contracture includes positioning, splinting, stretching, massage, pressure therapy, and passive and active exercises [5]. In this case report, all the therapeutic interventions showed significant results and helped the patient to regain functional activities.

## Conclusions

The early physiotherapy intervention in case of post-electric burn injury is efficient and has shown remarkable improvement in the ability of the patient to participate in activities of daily living with ease. The physiotherapeutic intervention focuses on preventing secondary complications post burn injury, which hampers the patient’s daily routine. This case report presents a well-designed comprehensive treatment regime and home care program in the patient's best interest.
